# Wubeizi Ointment Suppresses Keloid Formation through Modulation of the mTOR Pathway

**DOI:** 10.1155/2020/3608372

**Published:** 2020-09-30

**Authors:** Zhiming Tang, Yi Cao, Jicun Ding, Xiaoxiang Zhai, Mengqing Jing, Mengmeng Wang, Lu Lu

**Affiliations:** ^1^The First Clinical Medical College, Zhejiang Chinese Medical University, Hangzhou, China; ^2^Department of Dermatology, Xuzhou Hospital Affiliated to Nanjing University of Traditional Chinese Medicine, Xuzhou, China; ^3^Department of Dermatology, The First Affiliated Hospital of Zhejiang Chinese Medical University, Hangzhou, China; ^4^Department of Burns and Plastic Surgery, Xuzhou Central Hospital, Xuzhou, China; ^5^Department of Dermatology, Shanghai Seventh People's Hospital, Shanghai, China

## Abstract

**Background:**

Wubeizi (*Rhus chinensis* Mill.) ointment has been shown as an effective treatment for keloids. However, the protective mechanisms of Wubeizi ointment are not fully understood. The mammalian target of rapamycin (mTOR) has been demonstrated to be associated with keloid pathogenesis. In the present study, we investigated if Wubeizi ointment suppressed keloid formation through the modulation of key molecules of the rapamycin (mTOR) pathway including phosphatase and tensin homolog (PTEN), phosphatidylinositol 3-kinase (PI3K), and protein kinase B (Akt).

**Methods:**

A keloid mouse model and human keloid-derived fibroblasts were developed and treated with Galla chinensis. Immunohistochemistry, western blot, and reverse transcription-PCR were used to detect PI3K, PTEN, Akt, and mTOR in keloid tissues and keloid fibroblasts. The apoptosis and proliferation rate of keloid fibroblasts was, respectively, analyzed by flow cytometry according to the MTT assay. Statistical analysis was done using SPSS version 20.0. For two variable comparisons, a two independent samples *t*-test was used. For multiple variable comparisons, data were analyzed by one-way analysis of variance (ANOVA) followed by pairwise *q*-tests.

**Results:**

Our in vivo and in vitro studies showed that Wubeizi ointment suppressed keloid formation through inhibition of fibroblast proliferation and promotion of fibroblast apoptosis. The underlying basis involves downregulation of p-Akt and p-mTOR as well as upregulation of PTEN.

**Conclusion:**

These findings may contribute to a better understanding of the mechanisms of Wubeizi ointment for treating keloids.

## 1. Introduction

Keloids are formed because of abnormal overgrowth of scar tissue during the healing of skin wounds and are characterized by the proliferation of dermal fibroblast and the aberrant accumulation of extracellular matrix (type I and III) collagens, mucins, and glycosaminoglycans [1]. Wubeizi ointment has been shown as an effective treatment for keloids [1]. Our previous studies have demonstrated that Wubeizi ointment prevents the proliferation of the keloid-derived fibroblasts in a time- and dose-dependent manner [1]. In addition, Wubeizi ointment downregulated the expression of type I and III procollagen in keloid fibroblast and induced an S-phase arrest of keloid fibroblast [1, 2]. However, the mechanisms underlying the inhibitory effect of Wubeizi ointment on keloid formation are not fully understood.

mTOR is a serine/theronine kinase which plays an important role in the regulation of metabolic processes and translation rates. mTOR as a regulator of collagen expression and inhibition of mTOR reduced extracellular matrix deposition. Tissue extracts obtained from keloid scar showed an increase in the expression of mTOR, p70KDa S6 kinase, and their activated forms, suggesting an activation of mTOR in keloid scars [3]. The expression of transforming growth factor-*β* (TGF-*β*1), PI3K, Akt, and mTOR was significantly increased in pathological scar fibroblasts than in normal skin tissue or fibroblasts [4]. In addition, application of rapamycin to monoculture keloid fibroblasts could downregulate the expression of cytoplasmic proliferating cell nuclear antigen (PCNA), cyclin D1, fibronectin, collagen, and alpha-smooth muscle actin (alpha-SMA) in a time- and dose-dependent manner, suggesting the antiproliferative effect of rapamycin in the treatment of keloid scars [4]. Therefore, we hypothesized the possibility that Wubeizi ointment could act via the mTOR signaling pathway to inhibit the formation of keloid scar.

In the present study, we investigated the effect of Wubeizi ointment on key molecules of the mTOR pathway including PTEN, PI3K, and Akt. The results may contribute to a better understanding of the protective effect of Wubeizi ointment against keloid pathogenesis.

## 2. Material and Methods

### 2.1. Drug Preparation

The formula of Wubeizi ointment includes Chinese herbs *Salvia miltiorrhiza*, Clematis, black vinegar, *Galla Chinensis*, Cortex Lycii, and alum. It was prepared as described previously [1]. Briefly, the 7 ingredients were extracted first with water. After concentrating and cooling in a rotary evaporator, ethanol was slowly added, and the solution was stirred. The medicinal solution was refrigerated for 24-48 hours and filtered to obtain the Wubeizi medicinal solution. The concentration of the Wubeizi medicinal solution was adjusted to 50 mg/ml and 30 mg/ml to obtain a concentration of 5% and 3% Wubeizi medicinal solution, respectively, and stored at -20°C for future use. The Wubeizi ointment cream was prepared as follows: 50 g of white petroleum jelly, 20 g of cetyl alcohol, 20 g of stearyl alcohol, 100 g of stearic acid, 75 g of glyceryl monostearate, and 20 g of lanolin were mixed and heated to maintain a temperature of 80°C; 10 g of borneol, Nipal 1 g of gold ethyl ester, 8 g of triethanolamine, 5 g of Tween-80, 1 g of methyl paraben, 50 g of glycerol, and add 100 mL of purified water were added, heated to dissolve and maintained the temperature at 80°C, and slowly added to the oil phase with continuous stirring till it emulsified to form a matrix. Then, 19 ml of Wubeizi medicinal solution (3% or 5%) was added and stirred to room temperature to obtain the Wubeizi ointment cream of the corresponding concentration.

### 2.2. Specimen Source

The keloid scar tissues were obtained from the Department of Dermatology of Xuzhou Hospital affiliated to Nanjing University of Traditional Chinese Medicine (Jiangsu, China). The tissues were taken from the anterior part of chest after surgical resection for treatment, from 18 Chinese patients (8 males and 10 females with an average age of 22.8 ± 3.2, average course of disease of 2.1 ± 0.8 years). The normal skin tissue was resected near the keloid scar tissue.

### 2.3. Animal Models

Eighteen nude mice were purchased from the animal facility of Xuzhou Medical University. They were housed under conditions of constant temperature and humidity and with a 12 h light–12 h dark cycle. Keloid models were generated as described previously [5]. Briefly, the mice were anesthetized with an intraperitoneal injection of 0.1%pentobarbital sodium at 0.1 ml/10 g of body weight. When the mice were completely unconscious, human keloid scar fragments (8 mm × 5 mm × 5 mm) with epidermal and dermal tissue were implanted into the back of the nude mice like a full thickness skin graft. The animal models were ready for use after 14 days of implantation since by this time peripheral vascularization followed by anastomosis would have been completed [6]. The animals were then divided into three groups; i.e., control group, 3% Wubeizi ointment-treated group, and 5% Wubeizi ointment-treated group (*n* = 6 for each group). For the Wubeizi ointment-treated group, the corresponding concentration of Wubeizi ointment was applied to the skin (0.5 g/10 mm^2^, 3 times a day). For the control group, only the matrix of Wubeizi ointment was applied to the skin (0.5 g/10 mm^2^, 3 times a day).

After 30 days of Wubeizi ointment treatment, mice were anesthetized by intraperitoneal injection of 0.1% pentobarbital sodium at 0.1 ml/10 g of body weight and then killed by cervical dislocation, and the keloid tissues were disassociated. The size of the keloid tissues was measured by a vernier caliper as described previously [6].

### 2.4. Culture of Fibroblasts

The primary cells of the human keloid-derived fibroblasts were disassociated and cultured according to our published procedure [1]. Briefly, after rinsing in D-Hank's liquid (Shanghai Kang Lang Biological Technology Co., Ltd., Shanghai, China), keloid tissues were cut into small pieces (1 mm^3^) and placed into a flask. Subsequently, 5 ml of 0.25% trypsin solution (Shanghai Bioleaf Biotech Co., Ltd., Shanghai, China) was added into the flask. After 14 hours of digestion at 4°C, protease was removed, and 5 ml of complete media containing 10% fetal bovine serum (FBS, Shanghai Bioleaf Biotech Co., Ltd.) was added into the flask. Single cell suspension was prepared. Cells were then subcultured in a 25 ml flask and incubated in an incubator (Binder GmbH, Tuttlingen, Germany) at 37°C with 5% CO_2_. The generation was passaged every 2–3 days. The 3rd generation cells at logarithmic growth phase were analyzed.

Keloid fibroblasts were divided into four groups; i.e., control group, IGF-1-treated group, IGF-1+Wubeizi ointment-treated group, and Wubeizi ointment-treated group. For the control group, 20 *μ*l DMEM containing 5% FBS was added to the culture medium. For the IGF-1-treated group, 20 *μ*l IGF-1 (Thermo Fisher Scientific, USA) was added. IGF-1 is an agonist of the mTOR signaling pathway. For the IGF-1+Wubeizi ointment-treated group, 20 *μ*l mixture of IGF-1 and Wubeizi ointment was added. For the Wubeizi ointment-treated group, 20 *μ*l of the Wubeizi ointment solution was added. The Wubeizi ointment solution used for in vitro experiments was prepared by dissolving the extract of Wubeizi ointment in water at a concentration of 0.5 mg/ml.

### 2.5. Immunohistochemistry

Immunocytochemistry was conducted as described previously [6]. Briefly, keloid fragments (5 mm × 5 mm × 5 mm) were fixed in 4% paraformaldehyde at 4°C for 48 h, embedded in paraffin, sectioned at 4 *μ*m thickness by Rotary Paraffin Microtome Slicer paraffin embedding machine (Media Cybernetics, USA), and stained with hematoxylin and eosin for routine examination. Subsequently, the keloid sections were incubated with antibodies against p-PI3K (1 : 100, mouse anti-human PI3K monoclonal antibody, Santa Cruz, CA, USA), PTEN (1 : 100, mouse anti-human PTEN (a2b1) monoclonal antibody, Santa Cruz, CA, USA), p-Akt (1 : 100, mouse anti-human Akt monoclonal antibody, Santa Cruz, CA, USA), or p-mTOR (1 : 100, mouse anti-human mTOR monoclonal antibody, Santa Cruz, CA, USA). Bound antibodies were visualized using 3,3′-diaminobenzidine (DAB) as a chromogen (ZSGB Bio Co., Ltd, Beijing, China), and the slides were counterstained with hematoxylin. The positive expression of p-PI3K, PTEN, p-Akt, and p-mTOR was evaluated in four randomly selected fields under a light microscope (Olympus Corp., Tokyo, Japan). The mean optical densities (MOD) of positive expression were quantified with the Image Pro-Plus image analysis system (Media Cybernetics, Inc., Rockville, MD, USA).

### 2.6. Quantitative Real-Time PCR (qPCR)

The mRNA levels of PI3K, PTEN, Akt, and mTOR in the keloid tissues and cultured keloid fibroblasts were analyzed as described previously [4]. Briefly, total RNA was extracted from tissue lysate and fibroblasts using a RT-PCR kit (Western Co., Ltd., Chongqin, China) according to manufacturer's instructions. RNA content and its purity were examined with UV spectrophotometry, and the integrity of RNA was observed using formaldehyde gel electrophoresis. RNA was reverse transcribed to cDNA under the following reaction conditions: 25°C for 10 min, 42°C for 60 min, and 85°C for 5 min. Quantitative fluorescence PCR (Bio-Rad Laboratories, Inc., California, USA) was used to amplify the PIK3CA, Akt1, PTEN, and mTOR gene products. The reaction conditions were as follows: 94°C for 4 min, 94°C for 20 sec, 60°C for 30 sec, and 72°C for 5 min in 30 cycles. The primer sequences used are presented in Table 1. The grey values were quantified with the gel imaging analysis system (GDS8000, UVP, USA). Relative quantity (RQ) was calculated according to the equation 2^−*ΔΔ*Ct^. Each RT-qPCR experiment was repeated three times.

### 2.7. Western Blot

Western blot was performed as described previously [4]. Briefly, homogenate of the keloid tissues was prepared and centrifuged at 12,000 × g at 4°C for 15 min. The homogenate of cultured keloid fibroblasts was prepared and centrifuged at 15,000 × g at 4°C for 10 min. Protein in the supernatant of tissue homogenate was quantified using the Bradford method while that in the cultured fibroblast lysate was quantified using Lowry's method. Protein sample (50 *μ*g) was separated on 15% SDS-polyacrylamide gel, transferred to nitrocellulose membrane, and blocked with 5% skim milk powder for 2 hours at room temperature. Membranes were incubated with primary antibody against PI3K (1 : 500,sc-365290, Santa Cruz, CA, USA), p-PI3K (1 : 500,sc-56938, Santa Cruz, CA, USA), PTEN (1 : 500, sc-7974, Santa Cruz, CA, USA), Akt (1 : 500, sc-81434, Santa Cruz, CA, USA), p-Akt (1 : 500, sc-377556, Santa Cruz, CA, USA), mTOR (1 : 500, sc-517464, Santa Cruz, CA, USA), p-mTOR (1 : 500,sc-293089,Santa Cruz, CA, USA), and actin (1 : 2000,sc-8432,Santa Cruz, CA, USA) overnight at 4°C, washed with PBS, and then incubated with secondary antibody (biotinylated goat anti-rabbit secondary antibody, 1 : 1000, Berseebio Co., Ltd, Beijing, China) and conjugated to horseradish peroxidase for 1 h at room temperature. The membranes were washed with PBS, and immunoreactivity was visualized on Odyssey infrared fluorescence imager (LI-COR Biosciences, Lincoln, NE, USA). Images were analyzed by using the Image Pro-Plus image analysis system.

### 2.8. MTT Assay

The proliferation rate of keloid fibroblasts at specific time points (12 h, 24 h, 36 h and 48 h after drug treatment) was measured by the MTT assay. The MTT assay was performed as described previously [1]. Briefly, the cells were seeded in 96-well plates. After drug treatment (5% Wubeizi ointment), 20 *μ*l MTT was added to each well. After incubating for 4 h at 37°C, the liquid was removed, and dimethyl sulfoxide (150 *μ*l/well) was added. After dissolution of the blue-purple formazan, optical density (OD) was measured 1 h later in a microplate reader (BioRad, USA) at a wavelength of 490 nm.

### 2.9. Flow Cytometric Analysis

The apoptosis of keloid fibroblasts was analyzed by flow cytometry according as described previously [1]. Briefly, 48 h after drug treatment (5% Wubeizi ointment), keloid fibroblasts were collected and rinsed thrice in PBS. 5 *μ*l of Annexin V-FITC was added to 2 × 10^5^ cells for 15 min followed by 5 *μ*l PI and incubated in dark at 4°C for 5 min. The cells were subjected to flow cytometry (Beckman Coulter, Inc., USA). Dot intensities in both Q2 and Q3 regions indicated the number of apoptotic cells.

### 2.10. Statistical Analysis

Statistical analysis was done using SPSS version 20.0 (IBM SPSS Statistics for Windows, Armonk, NY: IBM Corp.). Data were presented as mean ± S.D. For two variable comparisons, a two independent samples *t*-test was used. For multiple variable comparisons, data were analyzed by the *χ*^2^ test or one-way analysis of variance (ANOVA) followed by pairwise *q*-tests.

## 3. Results

### 3.1. The Inhibitory Effect of Wubeizi Ointment on the Size of Keloid Scar

**Figure 1:**
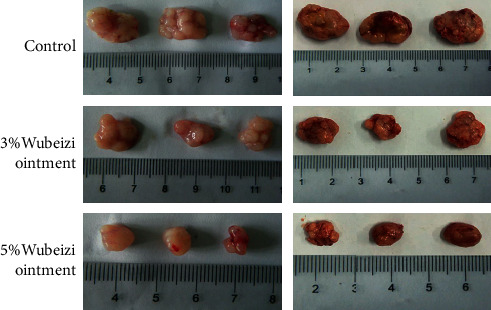
The inhibitory effect of Wubeizi ointment on the size of keloid scar. After 30 days of Wubeizi ointment treatment, mice were sacrificed, and the keloid tissues were disassociated. The size of the keloid tissues was measured by a vernier caliper. Representative pictures are shown (*n* = 6).

As show in Figure 1, the sizes of keloid tissues disassociated from 5% Wubeizi ointment-treated nude mice were 252.84 ± 38.57 mm^3^ (*n* = 6), which were significantly smaller than the sizes of keloid tissues disassociated from 3% Wubeizi ointment-treated nude mice (463.37 ± 51.82 mm^3^, *n* = 6) and control nude mice (785.63 ± 78.24 mm^3^, *n* = 6) (*P* < 0.05).

### 3.2. The Inhibitory Effect of Wubeizi Ointment on the Phosphorylated Protein and mRNA Levels of PI3K, PTEN, Akt, and mTOR in Keloid Tissues

**Figure 2:**
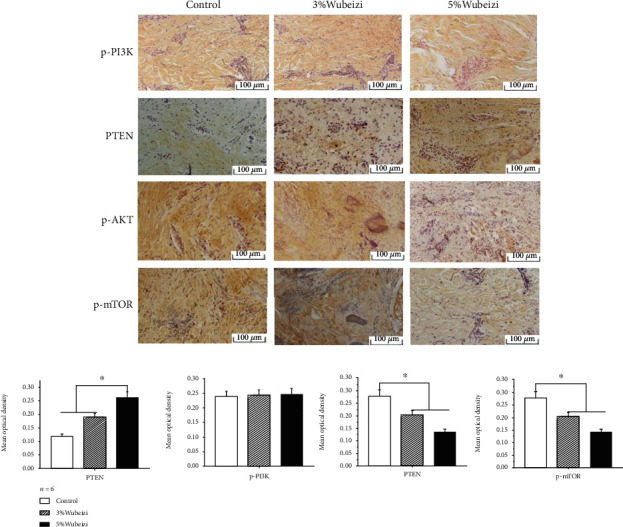
The inhibitory effect of Wubeizi ointment on the expression in keloid tissues. After 30 days of Wubeizi ointment treatment, mice were sacrificed, and the keloid tissues were disassociated. The expression of p-PI3K, PTEN, p-Akt, and p-mTOR in keloid tissues was detected by immunohistochemistry. Representative pictures are shown (×200). The MOD was quantified with the Image Pro-Plus image analysis system. MOD values were presented as *mean* ± *SD*. ^∗^*P* < 0.05, *n* = 6.

The results of immunohistochemistry showed that most of the p-PI3K, p-Akt, and p-mTOR were expressed in cytoplasm and a small amount of p-PI3K, p-Akt, and p-mTOR was observed in nucleus (Figure 2). In contrast, most of PTEN was expressed in nucleus, and a small amount was observed in cytoplasm (Figure 2). Compared to the control group, the expression of p-Akt and p-mTOR significantly decreased in keloid tissues from the 3% and 5% Wubeizi ointment-treated group, and the effect was dose-dependent (Figure 2, *P* < 0.05). In contrast, compared to the control group, the expression of PTEN significantly increased in keloid tissues from the 3% and 5% Wubeizi ointment-treated group, and the effect was dose-dependent (Figure 2, *P* < 0.05). However, there was no significant difference between the Wubeizi ointment-treated and control group in the expression of p-PI3K in keloid tissues (Figure 2, *P* = 0.735).

**Figure 3:**
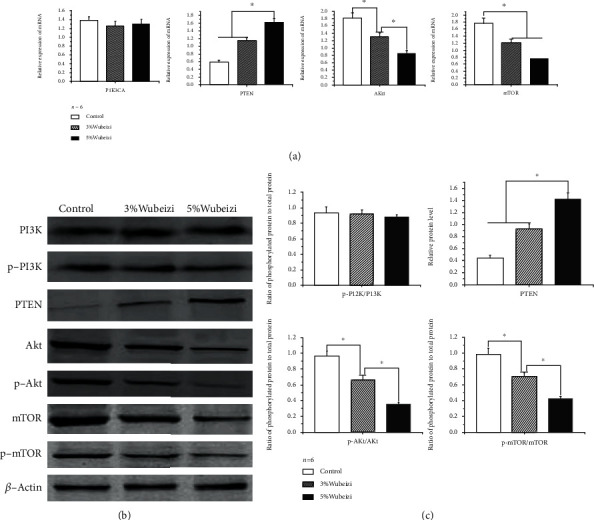
The effect of Wubeizi ointment on the mRNA and protein levels in keloid tissues. (a) The inhibitory effect of Wubeizi ointment on the mRNA levels of PIK3CA, PTEN, Akt1, and mTOR in keloid tissues. After 30 days of Wubeizi ointment treatment, mice were sacrificed, and the keloid tissues were disassociated. The mRNA levels of PIK3CA, PTEN, Akt1, and mTOR in keloid tissues were measured by qPCR. The grey values were quantified with the gel imaging analysis system. The RQ of mRNA was calculated and presented as *mean* ± *SD*. ^∗^*P* < 0.05, *n* = 6. (b) The inhibitory effect of Wubeizi ointment on the protein levels of p-PI3K, PTEN, p-Akt, and p-mTOR in keloid tissues. After 30 days of Wubeizi ointment treatment, mice were sacrificed, and the keloid tissues were disassociated. The protein levels of p-PI3K, PTEN, p-Akt, and p-mTOR in keloid tissues were determined by western blot. Representative pictures are shown. Band intensities were quantified with the Image Pro-Plus image analysis system. The relative ratios of proteins to actin band density were presented as *mean* ± *SD*. ^∗^*P* < 0.05, *n* = 6.

The results of qPCR and western blot showed that, compared to the control group, mRNA of Akt1 (Figure 3(a)), and phosphorylated protein levels of Akt, mTOR significantly decreased in keloid tissues from the Wubeizi ointment-treated group (Figure 3(b)), and the effect is dose-dependent (*P* < 0.05). In contrast, compared with the control group, mRNA and protein levels of PTEN were significantly increased in keloid tissues from the Wubeizi ointment-treated group, and the effect is dose-dependent (Figures 3(a) and 3(b), *P* < 0.05). However, there were no significant differences found between the Wubeizi ointment-treated and control group in the mRNA of PIK3CA and phosphorylated protein levels of PI3K in keloid tissues (Figures 3(a) and 3(b), *P* > 0.05).

These data suggested that Wubeizi ointment could downregulate the mRNA expression levels of Akt1 and mTOR and upregulate PTEN mRNA expression levels in keloid tissues. It also could reduce phosphorylated protein levels of Akt and mTOR in keloid tissues.

### 3.3. The Inhibitory Effect of Wubeizi Ointment on Proliferation and Apoptosis of Keloid Fibroblast

**Figure 4:**
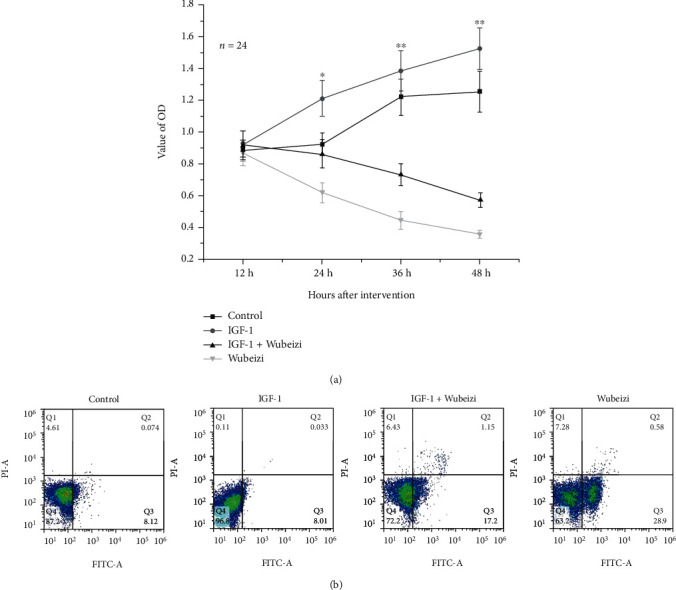
The effect of Wubeizi ointment on fibroblast proliferation and apoptosis. (a) The inhibitory effect of Wubeizi ointment on fibroblast proliferation. After IGF-1 and/or Wubeizi ointment treatment, the proliferation of keloid fibroblasts at specific time points was detected by the MTT assay. OD valves were read on an enzyme-linked immunoassay analyzer and presented as *mean* ± *SD*. ^∗^*P* < 0.05; ^∗∗^*P* < 0.01, *n* = 24. (b) The effect of Wubeizi ointment on fibroblast apoptosis. After IGF-1 and/or Wubeizi ointment treatment, the apoptosis of keloid fibroblasts was detected by flow cytometry. Representative pictures are shown. Dot intensities in both Q2 and Q3 regions indicated the number of apoptotic cells.

Results of the MTT assay showed that IGF-1 promoted fibroblast proliferation whilst Wubeizi ointment inhibited fibroblast proliferation (Figure 4(a), *P* < 0.05). In addition, the inhibitory effect of Wubeizi ointment on fibroblast proliferation could be attenuated by IGF-1 (Figure 4(a)). Results of flow cytometry showed that Wubeizi ointment was able to promote fibroblast apoptosis (control vs. Wubeizi ointment-treated group, 8.20 ± 0.78 vs. 29.82 ± 2.34, *n* = 24, *P* < 0.05) (Figure 4(b)). The inhibitory effect of Wubeizi ointment on fibroblast apoptosis could be attenuated by IGF-1 (Wubeizi ointment-treated vs. IGF-1+Wubeizi ointment-treated group, 29.82 ± 2.34 vs. 18.47 ± 1.86, *n* = 24) (Figure 4(b)).

### 3.4. The Inhibitory Effect of Wubeizi Ointment on the Phosphorylated Protein and mRNA Levels of PI3K, PTEN, Akt, and mTOR in Keloid Fibroblasts

**Figure 5:**
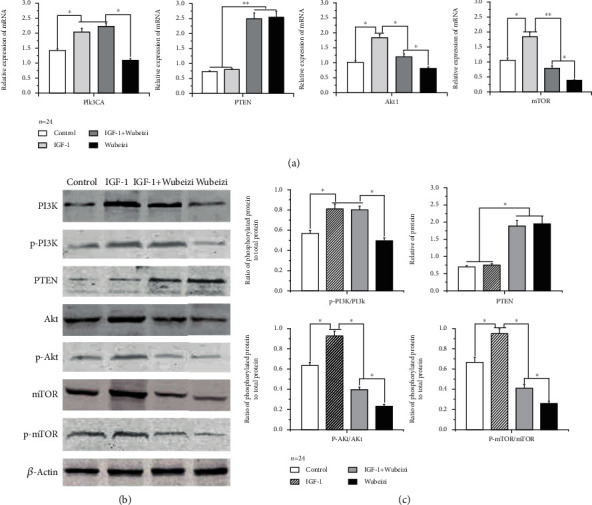
The effect of Wubeizi ointment on the expression of PI3K, PTEN, Akt, and mTOR. (a) The inhibitory effect of Wubeizi ointment on the mRNA levels of PIK3CA, PTEN, Akt1, and mTOR in keloid fibroblasts. After IGF-1 and/or Wubeizi ointment treatment, the mRNA levels of PIK3CA, PTEN, Akt1, and mTOR in keloid fibroblasts were measured by qPCR. The grey values were quantified with the gel imaging analysis system. The RQ of mRNA were calculated and presented as *mean* ± *SD*. ^∗^*P* < 0.05; ^∗∗^*P* < 0.01, *n* = 24. (b) The inhibitory effect of Wubeizi ointment on the protein levels of p-PI3K, PTEN, p-Akt, and p-mTOR in keloid fibroblasts. After IGF-1 and/or Wubeizi ointment treatment, the protein levels of p-PI3K, PTEN, p-Akt, and p-mTOR in keloid fibroblasts were determined by western blot. Representative pictures are shown. Band intensities were quantified with the Image Pro-Plus image analysis system. The relative ratios of proteins to actin band density were presented as *mean* ± *SD*. ^∗^*P* < 0.05; ^∗∗^*P* < 0.01, *n* = 24.

The results of qPCR showed that, compared to the control group, mRNA levels of PIK3CA, Akt1, and mTOR were significantly increased in keloid fibroblasts from the IGF-1-treated group (Figure 5(a), *P* < 0.05).

Compared to the control and Wubeizi ointment treated-group, mRNA levels of PIK3CA were significantly increased in keloid fibroblasts from the IGF-1+Wubeizi ointment-treated group and IGF-1-treated group (Figure 5(a), *P* < 0.05). However, there was no significant difference between the Wubeizi ointment-treated and control group in PI3K mRNA levels in keloid fibroblasts (Figure 5(a), *P* = 0.418). Compared to the control and IGF-1-treated group, mRNA levels of PTEN were significantly increased in keloid fibroblasts from the IGF-1+Wubeizi ointment-treated group and Wubeizi ointment-treated group (Figure 5(a), *P* < 0.01). However, there was no significant difference between the IGF-1-treated and control group in the mRNA levels of PTEN in keloid fibroblasts (Figure 5(a), *P* > 0.05). In contrast, compared to the control group, mRNA levels of Akt1 and mTOR were significantly decreased in keloid fibroblasts from the IGF-1+Wubeizi ointment-treated group and Wubeizi ointment-treated group (Figure 5(a), *P* < 0.05). The mRNA levels of Akt1 and mTOR in keloid fibroblasts from the IGF-1+Wubeizi ointment-treated group was higher than those in keloid fibroblasts from the Wubeizi ointment-treated group (Figure 5(a), *P* < 0.05).

The results of western blot showed that, compared to the control group, phosphorylated protein levels of p-PI3K, p-Akt, and p-mTOR were significantly increased in keloid fibroblasts from the IGF-1-treated group (Figure 5(b), *P* < 0.05).

Compared to the control and Wubeizi ointment-treated-treated group, protein levels of p-PI3K were significantly increased in keloid fibroblasts from the IGF-1+Wubeizi ointment-treated group and IGF-1-treated group (Figure 5(b), *P* < 0.05). However, no significant difference was found between the control and Wubeizi ointment-treated-treated group in the protein levels of p-PI3K in keloid fibroblasts (Figure 5(b), *P* > 0.05). Compared to the control and IGF-1-treated group, protein levels of PTEN were significantly increased in keloid fibroblasts from the IGF-1+Wubeizi ointment-treated group and Wubeizi ointment-treated group (Figure 5(b), *P* < 0.05). No significant difference was found between the control and IGF-1 group in the protein levels of PTEN in keloid fibroblasts (Figure 5(b), *P* = 0.364). In contrast, compared to the control group, protein levels of p-Akt and p-mTOR were significantly decreased in keloid fibroblasts from the IGF-1 + Wubeizi ointment-treated group and Wubeizi ointment-treated group (Figure 5(b), *P* < 0.05). The protein levels of p-Akt and p-mTOR in keloid fibroblasts from the IGF-1+Wubeizi ointment-treated group was higher than those in keloid fibroblasts from the Wubeizi ointment-treated group (Figure 5(b), *P* < 0.05).

These data suggest that Wubeizi ointment was able to downregulate the mRNA expression levels of Akt and mTOR and upregulate the mRNA expression levels of PTEN in keloid fibroblasts. It also could reduce phosphorylated protein levels of Akt and mTOR in keloid fibroblasts. These effects of Wubeizi ointment could be attenuated by IGF-1.

## 4. Discussion

The current study explored if Wubeizi ointment could suppress keloid formation through the modulation of the mTOR signaling pathway. The results showed Wubeizi ointment could reduce the size of keloid morphologically in nude mice and could influence the expression pattern of some key factors involved in the mTOR pathway both in the keloid tissues and in cultured keloid fibroblast cells. While there was upregulation of PTEN and downregulation of p-Akt and p-mTOR, no significant difference in the expression level of p-P13K was seen.

Keloids are formed due to abnormal cellular proliferation of fibroblasts producing high amounts of collagen and matrix metalloproteinases (MMP). Aberrant accumulation of extracellular matrix collagens, mucins, and glycosaminoglycans because of an imbalance between collagen production and degradation of the extracellular matrix along can lead to the elevated production of cytokines and proinflammatory responses. Faulty fibroblast regulation resulting in proliferation of fibroblasts and apoptosis inhibition plays an important role in the keloid development and scar formation [7].

Our previous study demonstrated that Wubeizi ointment increased fibroblasts at the S phase thereby greatly reduced dividing fibroblasts in addition to inhibiting the proliferation of keloid-derived fibroblasts in a time- and dose-dependent manner [1]. Moreover, Wubeizi ointment could downregulate the expression of type I and III procollagen in keloid fibroblast, subsequently reducing collagen deposition in the keloid tissue [2]. In support of these findings, the present study demonstrated that Wubeizi ointment reduced the size of the keloid tissue through inhibition of fibroblast proliferation and promotion of fibroblast apoptosis.

It has been demonstrated that the proliferation of keloid fibroblasts could be regulated by various cytokines, such as TGF-*β*1 [4]. Keloid fibroblast proliferation and transdifferentiation are promoted by TGF-*β*1 through upregulation of the miR-21 and PTEN/AKT signaling pathway [8]. Keloid fibroblast proliferation and collagen synthesis as well as inhibition of the collagen-degrading activity of MMP are stimulated by TGF-*β*. Scarring can be reduced in rodents by inhibition of TGF-*β* signaling proteins [9]. The mTOR signaling pathway plays an important role in the TGF-*β*1 signal transduction [7–12]. The mTOR signal pathway includes upstream PI3K, Akt, and PTEN, among which PTEN acts as a negative feedback regulating factor of the mTOR signal pathway [8]. The mTOR gene is closely associated with the occurrence of various tumors, such as glioma, esophageal, and colorectal cancer [13–19]. Pathological characteristics of keloid are very similar to those of benign solid tumor and can invasively grow to surrounding normal tissues [4]. These findings suggest that the mTOR signaling pathway may be important in the process of keloid fibroblast proliferation, and out study provides evidence that genes involved in this process are modulated by Wubeizi ointment.

It was found that inhibition of mTOR can reduce extracellular matrix deposition [20, 21]. In addition, the expression of mTOR and its upstream molecules PI3K and Akt were significantly increased in keloid tissues [3, 4]. Furthermore, application of rapamycin to monoculture keloid fibroblasts could downregulate the expression of cytoplasmic PCNA, cyclin D1, fibronectin, collagen, and alpha-SMA [3]. These data raise the possibility that Wubeizi ointment inhibits the formation of keloid scar through modulation of the mTOR pathway. To confirm the hypothesis, in vitro experiments with cultured keloid fibroblasts and in vivo experiments with nude mice induced with keloids were performed, and the results showed that Wubeizi ointment can interfere with the mTOR signaling pathway. We used IGF-1, an agonist of the mTOR signaling pathway [19], to activate the mTOR signaling pathway after adding Wubeizi ointment, and then the proliferation of keloid fibroblasts was detected to test whether the drug efficacy is inhibited. This can confirm whether the mechanism of Wubeizi ointment regulates the mTOR signaling pathway to inhibit the proliferation of keloid fibroblasts. In the in vitro experiment, after the addition of IGF-1, the effect of Wubeizi ointment on inhibiting proliferation of keloid fibroblasts and promoting its apoptosis was diminished. It suggests that the mechanism of Wubeizi ointment inhibiting the proliferation of keloid fibroblasts is related to the regulation of the mTOR signaling pathway. This study demonstrated that Wubeizi ointment inhibited the proliferation of keloid fibroblasts and promoted the apoptosis of keloid fibroblasts, probably through downregulation of Akt and mTOR and upregulation of PTEN. This study is limited by the fact that it is preliminary in nature, and in-depth mechanism of action of Wubeizi ointment is warranted in future studies.

## 5. Conclusion

In conclusion, the present study investigated if Wubeizi ointment inhibited the keloid formation through modulation of key molecules of the mTOR pathway including PTEN, PI3K, and Akt. The results of the in vivo and in vitro studies suggested that Wubeizi ointment inhibited the proliferation of keloid fibroblasts and promoted the apoptosis of keloid fibroblasts, probably through downregulation of Akt and mTOR and upregulation of PTEN. These findings may contribute to a better understanding of the mechanism of Wubeizi ointment for treating keloid and provide a theoretical basis for its clinical application.

## Figures and Tables

**Table 1:** Primer for real-time PCR.

Primer	Sequences (5′->3′)
PI3K3CA-forward	TGGATGCTCTACAGGGCTTT
PI3K3CA-reverse	GTCTGGGTTCTCCCAATTCA
PTEN-forward	TTGAAGACCATAACCCACCA
PTEN-reverse	CACATAGCGCCTCTGACTGG
Akt1-forward	TCTATGGCGCTGAGATTGTG
Akt1-reverse	CTTAATGTGCCCGTCCTTGT
mTOR-forward	ACTCGCTTCTATGACCAACTGA
mTOR-reverse	TTTCCATGACAACTGGGTCATTG
*β*-Actin-forward	CCTAGA AGCATTTGCGGTGG
*β*-Actin-reverse	GAGCTACGAGCTGCCTGACG

## Data Availability

The datasets used and/or analyzed during the current study are available from the corresponding author on reasonable request.
